# Factor and reliability analysis of a brief scale to measure motivation to change lifestyle for dementia risk reduction in the UK: the MOCHAD-10

**DOI:** 10.1186/s12955-019-1143-8

**Published:** 2019-05-02

**Authors:** Deborah Oliveira, Aimee Aubeeluck, Ed Stupple, Sarang Kim, Martin Orrell

**Affiliations:** 1 Department of Psychiatry, School of Medicine, Federal University of Sao Paulo (UNIFESP), Rua Major Maragliano, 241 - Predio Academico - Vila Mariana Mariana, São Paulo - CEP: 04017-030, São Paulo, SP Brazil; 2 School of Health Sciences, University of Nottingham, Nottingham, UK; 3 Human Sciences Research Centre, College of Life and Natural Sciences, University of Derby, Derby, UK; 4 Centre for Research on Ageing, Health and Wellbeing, Research School of Population Health, Australian National University, Canberra, Australia; 5 Wicking Dementia Research and Education Centre, University of Tasmania, Hobart, Australia; 6 Institute of Mental Health, Division of Psychiatry and Applied Psychology, School of Medicine, University of Nottingham, Nottingham, UK

**Keywords:** Dementia, Prevention, Risk reduction, Clinical tool, Scale development, Outcome measure, Validation, Psychometrics, Exploratory factor analysis, Confirmatory factor analysis

## Abstract

**Background:**

Modifying lifestyle risk factors for dementia is a public health priority. Motivation for change is integral to the modification of health-related risk behaviours. This study investigates the psychometric properties of the previously validated tool entitled ‘Motivation to Change Lifestyle and Health Behaviours for Dementia Risk Reduction Scale’ (MCLHB-DRR) for use in the UK.

**Methods:**

A sample of 3,948 individuals aged 50 and over completed the 27-item MCLHB-DRR online. The psychometric properties of the scale were explored via Exploratory Principal Axis Factoring (PAF) with Oblimin rotation. Confirmatory Factor Analysis (CFA) was used to confirm the factor structure using chi-square (χ2), the goodness-of-fit index (GFI), the comparative fit index (CFI), the root mean square error of approximation (RMSEA) and Root Mean Square Residual (RMR) as fit indices to evaluate the model fit. Internal consistency (Cronbach α) was measured for the final scale version.

**Results:**

Exploratory Factor Analysis (EFA) resulted in a parsimonious 10-item, two-factor structure (5 items each, factor loadings > 0.3) that explained 52.83% of total variance. Based on the Pattern Matrix, Factor 1 was labelled “Positive Cues to Action” and Factor 2 was labelled “Negative Cues to Action”. After addressing some errors in covariances, CFA showed a good fit where all fit indices were larger than 0.90 (GFI = 0.968, CFI = 0.938) and smaller than 0.08 (RMSEA = 0.072, RMR = 0.041). The standardized coefficients of Factor 1 and Factor 2 ranged from 0.30 to 0.73 and were all statistically significant (*p* < 0.001). The final scale showed moderate to high reliability scores (Factor 1 α = 0.809; Factor 2 α = 0.701; Overall α = 0.785).

**Conclusions:**

The new MOCHAD-10 (Motivation to Change Behaviour for Dementia Risk Reduction Scale) is a short, reliable and robust two-factor, 10-item clinical tool for use in preventative health care and research to evaluate motivation to change lifestyle for dementia risk reduction.

## Background

Recent evidence suggest that about 30% of all dementia cases could be prevented through the management of modifiable risk factors, such as obesity, diabetes, alcohol consumption, high blood pressure, and smoking [1, 2]. Delaying the onset of dementia by just one year is likely to reduce its prevalence by 11% by 2050, while delaying it by five years could halve the number of people living with dementia by 2050 [3]. Hence, public health actions aimed at reducing dementia risk should be a priority [4]. Improving individual lifestyles for better health status is a complex task that depends on the individual’s attitudes and beliefs towards health and illnesses [5]. Although some people respond to health risks through the adoption of health behaviours to reduce risks, this is often difficult to achieve as it encompasses an array of cognitive, social and emotional factors [5]. Moreover, individuals need to be motivated to change their behaviour and have the self-belief that they can do so [6]. It is therefore important to understand the individuals’ attitudes and motivation to make lifestyle changes in order that interventions targeted at dementia risk reduction can be designed and implemented effectively.

There are a number of theoretical perspectives and various studies that focus on behaviour change and lifestyle choices [7, 8]. For example, the behaviour change wheel proposed by Michie et al. [9] encompasses several behavioural change frameworks in one. It includes capability, opportunity and motivation for behaviour change, as well as intervention functions and policy categories which are necessary for the change to occur in ‘real life’. This model has been reliably used in public health actions for behaviour change in obesity and tobacco use, for example [9]. Moreover, Rothman and colleagues [10] argue that people who are motivated by their own needs and desires are more likely to initiate and sustain a new healthy behaviour. They also suggest that particularly in longitudinal behaviour change interventions, high levels of motivation are essential for initiation and sustained change. Nevertheless, across behaviour change frameworks per se, study results are often inconsistent and limit conclusions as to what makes for successful behaviour change and why individuals may not change their behaviour.

Behavioural change interventions aimed at reducing specific unhealthy behaviours can be effective [e.g. smoking cessation interventions [11] and blood pressure control interventions [12]]. However, similar single-domain interventions (focused on only one risk factor, such as smoking) aimed at modifying such risk factors for dementia risk reduction have mainly shown inconclusive results [13]. Conversely, a Finish 2-year multi-domain, longitudinal intervention aimed at modifying several lifestyle-related risk factors simultaneously (diet, physical exercise, cognitive training and vascular risk monitoring) was effective to prevent cognitive decline [14]. This indicates that population-attributable risk of each dementia risk factor should not be considered in isolation, but should be taken as a combined set [1].

Behaviour change for dementia risk reduction is likely to be influenced by some dementia-specific issues, such as worry or fear of dementia, as well as the individual perceived susceptibility to the condition [15]. Measurement tools to evaluate individual motivation to change lifestyle for dementia risk reduction should therefore be dementia-specific. Assessment of the psychometric properties of a measurement tool is essential to guarantee that the tool is able to measure the construct it proposes to measure in a consistent way across individuals and populations and over time [16]. The psychometric properties for a given tool (e.g. factor structure, reliability scores) may vary when this is applied in different cultures or age groups, for example. For this reason, it is important to assess the tool measurement properties again when using it in a different context from where it was initially developed to ensure that this is still fit for purpose [16]. In this study, we evaluated the factor structure and the reliability scores of the ‘Motivation to Change Lifestyle and Health Behaviours for Dementia Risk Reduction Scale’ (MCLHB-DRR) [5] to create a brief tool for the assessment of individual motivation to change lifestyle for dementia risk reduction for use in the UK. It is also anticipated that a shorter version of this scale is likely to be more readily used in clinical practice.

## Materials and methods

### Aim

To evaluate the factor structure and the reliability scores of the ‘Motivation to Change Lifestyle and Health Behaviours for Dementia Risk Reduction Scale’ (MCLHB-DRR) [5] to create a brief tool for the assessment of individual motivation to change lifestyle for dementia risk reduction.

### Design

An online cross-sectional survey of individuals aged 50 and over without a dementia diagnosis was conducted. The study is reported as per quality criteria proposed for measurement properties of health status questionnaires guidelines [17].

### Sample and recruitment

Individuals taking part in the study were aged 50 and over and had no self-reported dementia diagnosis. Potential participants were recruited via social media and paper adverts. The Join Dementia Research network (a UK-based online platform in which thousands of people with and without dementia are voluntarily registered to take part in dementia-related research, such as this survey) and the UK National Institute for Health Research (NIHR) Portfolio, were also utilised. Before completing the online survey, all potential participants confirmed that they met the study inclusion criteria and consented to take part. Participants took approximately 15 min to complete the anonymous survey. A non-probabilistic sample of 3948 individuals was enrolled.

### Variables

Demographic information included gender, age, ethnicity, marital status and employment status. The Motivation to Change Lifestyle and Health Behaviours for Dementia Risk Reduction Scale (MCLHB-DRR) [5] was developed in Australia. Using confirmatory factor analysis (CFA), the authors found a seven-factor solution containing 27 items in total. Internal consistency (α = 0.61–0.86) and test-retest reliability (α = 0.55 to 0.78) were questionable to high in all sub-scales. Measurement of invariance across gender and age was also established. The 27-item version of the scale is divided in the following sub-scales/domains of motivation: perceived susceptibility (4 items – e.g. ‘There is a strong possibility that I will develop dementia’), perceived severity (5 items – e.g. ‘The thought of dementia scares me’), perceived benefits (4 items – e.g. ‘I have a lot to gain by changing my lifestyle and health behaviour’), perceived barriers (4 items e.g. ‘Changing lifestyle and behaviour interferes with my schedule’), cues to action (4 items – e.g. ‘Being forgetful makes me think I have to change my lifestyle and behaviour’), general health motivation (4 items – e.g. ‘I think I have to pay attention to my own health’), and self-efficacy (2 items e.g. ‘I am able to make differences that will change the risk of developing dementia’).

### Ethics

This study was approved by the East Midlands - Nottingham Research Ethics Committee Ethics Committee (IRAS project ID 177280; REC reference 16/EM/0044). The study was conducted in accordance with the British Psychological Society Code of Ethics and all participant who took part in the anonymous survey provided informed consent.

### Data analysis

As participants were required to complete all the questions, the study had no missing data. The study sample was randomly split using participant entry codes; the first half was used to identify the measurement model with Exploratory Factor Analysis (EFA), and the second half was used to cross-validate the model using Confirmatory Factor Analysis (CFA). Best practices for conducting EFA and CFA were followed and are described below [18, 19]. We calculated the Kaiser-Meyer-Olkin (KMO) for checking sampling adequacy and the Bartlett’s Test of Sphericity to assess the suitability of the data for factor analysis [16]. Low off-diagonal values in the anti-image correlation matrix provided further evidence that the data were suitable for factor analysis [16].

For the EFA, first a total score was computed as the sum of ratings across all 27 items. Individual items were then correlated with the sum total of the scale and were excluded where r < 0.3 [20]. Principal Axis Factoring (PAF) with Oblimin rotation (Kaiser Normalization) was conducted on the remaining items. We observed the scree plot as a measure for factor extraction, as per recommended previously [18]. The Pattern Matrix was used for factor interpretation. We set a threshold for factor loadings based on Comrey & Lee [21] ‘fair’ criterion of 0.45 [16] and items not meeting this threshold were excluded. The remaining items from the EFA were then tested using CFA.

For the CFA, error covariances identified by modification indices were only examined further if they would reduce large residuals and significantly improve the fit of a poorly fitting model. Five types of fit indices were used to evaluate the model fit: the model chi-square (χ2), the goodness-of-fit index (GFI), the comparative fit index (CFI), the root mean square error of approximation (RMSEA) and Root Mean Square Residual (RMR). An acceptable model fit was defined as follows: *p*-value for the χ2 larger than 0.05, CFI and GFI values between 0.90 and 0.95 or above and RMSEA and RMR values of 0.08 or below. The reliability scores (internal consistency) of the final scale version was then analysed using Cronbach’s Alpha. The data was analysed using SPSS® 24 for the EFA, and AMOS® version 25 for the CFA.

## Results

### Sample

Participants were mostly women (*n* = 2880; 72.9%), from a White ethnic background (*n* = 3805; 97.1%) and living in England (*n* = 3586; 90.8%). The mean age was 62 (±8.0; range 50–93), over half (*n* = 2502; 63.4%) were married and almost half had a current job (*n* = 1958; 49.6%). Majority of participants were graduated or had a post-graduation degree (*n* = 2297; 58.2%) (Table 1).

**Table 1:** Demographic Information (*n* = 3948)

Variables	*n* (%)
Location	
England	3586 (90.8%)
Wales	126 (3.2%)
Northern Ireland	21 (0.5%)
Scotland	215 (5.4%)
Gender	
Female	2880 (72.9%)
Male	1060 (26.8%)
Other/Prefer not to say	8 (0.3%)
Age	
50–59	1587 (40.5%)
60–69	1554 (39.7%)
70+	771 (19.8%)
Highest qualification	
Non-graduate	1651 (41.8%)
Graduate and post-graduate	2297 (58.2%)
Relationship status	
Single	356 (9.0%)
Married	2502 (63.4%)
Civil partnership	54 (1.4%)
Separated/Divorced	407 (10.3%)
Widowed	245 (6.2%)
In a relationship	377 (9.5%)
Prefer not to say	7 (0.2%)
Do you currently have a job?	
Yes	1958 (49.6%)
No	1990 (50.4%)
What is your ethnic group?	
White	3835 (97.1%)
Others (Black/Asian/Arab/Other/Prefer not to say)	109 (2.8%)

### Exploratory factor analysis

The Kaiser-Meyer-Olkin (KMO) measure of sampling adequacy suggested that the sample was factorable (KMO = 0.815) and the Bartlett’s Test of Sphericity was highly significant (χ 2 = 5533, df = 45, *p* < 0.001). Results from EFA and scree plot indicated that 10 items loaded onto two factors should be retained. Therefore, the analysis was re-run specifying the extraction of two factors, which resulted in a parsimonious factorial structure explaining 52.83% of total variance (Table 2). No cross-loadings were identified. Five items loaded on to Factor 1. These items are related to health beliefs and therefore this Factor was labelled ‘Positive Cues to Action’. Five items loaded on to Factor 2 and were related to perceived severity. This Factor was labelled ‘Negative Cues to Action’. The retained items represent five of the seven domains of the Health Belief Model, except for ‘perceived barriers’ and ‘general health motivation’.

**Table 2:** Pattern matrix containing factor loadings (EFA) and respective theory domain represented by each retained in the MOCHAD-10

Retained items	Factor 1 Positive Cues to Action	Factor 2 Negative Cues to Action	Domains from the Health Belief Model^a^
1. I am able to make differences that will change the risk of developing dementia	0.743	–	CA/SE
2. Changing my lifestyle and health habits can help me reduce my chance of developing dementia	0.709	–	CA/PB
3. Having risk factor (s) for dementia makes me think I have to change my lifestyle and behaviour	0.649	–	CA
4. Learning more about dementia from the media makes me think I have to change my lifestyle and behaviour	0.643	–	CA
5. Knowing family member (s) with dementia makes me think I have to change my lifestyle and behaviour	0.535	–	CA
6. When I think about dementia my heart beats faster	–	0.774	CA/PSE
7. When I think about dementia I feel nauseous	–	0.729	CA/PSE
8. The thought of dementia scares me	–	0.594	CA/PSE
9. My feelings about myself would change if I develop dementia	–	0.377	CA/PSE
10. There is a strong possibility that I will develop dementia	–	0.374	CA/PSU

^a^*SE* self-efficacy, *PB* perceived benefits, *CA* cues to action, *PSE* perceived severity, *PSU* perceived susceptibility

### Confirmatory factor analysis

**Fig. 1:**
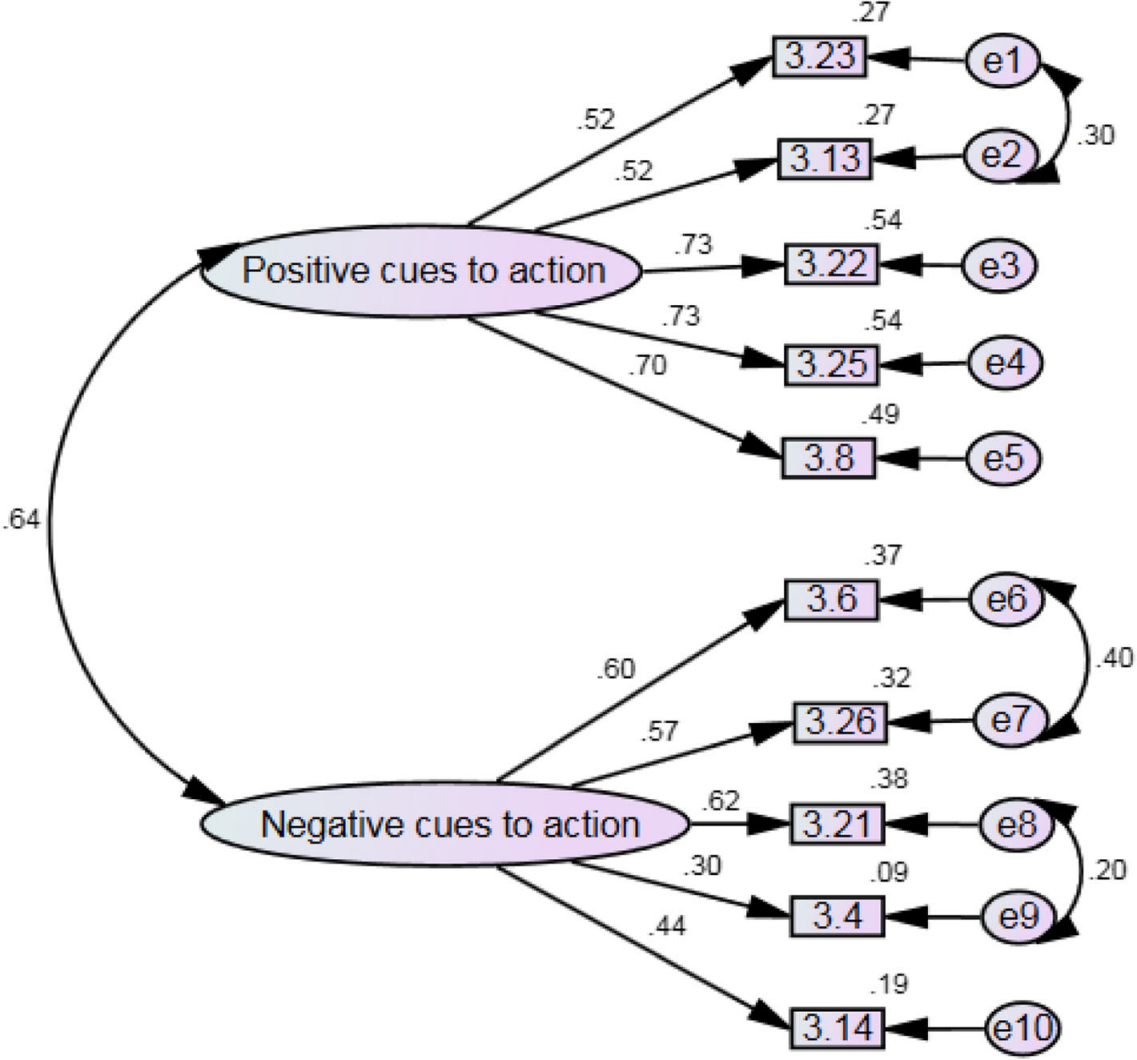
PRISMA Flow Diagram

CFA was implemented on the modified 10-item, two-factor model. The CFA initially suggested that this model was not a good fit of the data. The CFI and GFI were less than the accepted value of 0.9 and 0.95 respectively (CFI = 0.871, GFI = 0.932). The RMSEA (0.099) was also outside the accepted value of 0.08 or less. Error covariances were addressed and the model indicated a better fit, with all fit indices being larger than 0.90 (GFI = 0.968, CFI = 0.938) and smaller than 0.08 (RMSEA = 0.072, RMR = 0.041). χ2 was 344.4 (d.f. = 31, *p* < 0.001), where the low *p*-value was likely to be due to large sample size. The standardized coefficients of Factor 1 (Positive Cues to Action) (5 items) and Factor 2 (Negative Cues to Action) (5 items) ranged from 0.30 to 0.73 and were all statistically significant (*p* < 0.001) (Fig. 1).

### Reliability

Analysis of the internal consistency of the final two-factor, 10-item scale showed moderate to high reliability in both retained factors (Cronbach’s α = 0.809 for Factor 1 and Cronbach’s α = 0.701 for Factor 2). The full scale was also highly reliable (Cronbach’s α = 0.785).

## Discussion

This study reports on the factor structure and reliability scores of a scale to measure motivation to change lifestyle for dementia risk reduction. After carrying out both EFA and CFA in a large sample of middle-aged and older adults in the UK, the 27-item Australian version of the MCLHB-DRR was reduced to a short, robust and parsimonious two-factor 10-item scale (Factor 1 = Positive Cues to Action; Factor 2 = Negative Cues to Action), hereafter named ‘MOCHAD-10’ (Motivation to Change Behaviour for Dementia Risk Reduction). The two factors represent both positive and negative elements related to motivation to change lifestyle covering a range of beliefs and feelings. The overall internal consistency score for the MOCHAD-10 demonstrated that both retained factors are correlated, confirming that both subscales appear to be different facets of the same underlying construct. Five ‘perceived severity’ items were retained in the final 10-item model as part of the ‘Negative Cues to Action’ factor (Factor 2), demonstrating the role of fear of dementia in driving motivation to change lifestyle for dementia risk reduction.

The retained items might reflect findings from recent studies which have suggested a ‘panic-blame’ status within UK media coverage of dementia, with dementia being narrated as catastrophic and individuals living with dementia being blamed for having the disease [22]. This study sample was largely formed by women and older adults. Previous research has demonstrated that individuals with such characteristics tend to have more negative attitudes towards others living dementia when compared to men and younger individuals [23] and it may be possible that such attitudes would also translate to the self. This might explain why 4 out of the 5 items related to ‘perceived severity’ were retained in the UK model. Furthermore, most people in this study were reactive in terms of what they need to do to reduce their risk of having dementia as most items retained in the final model were related to ‘cues to action’. This corroborates findings from previous studies in which women and older adults are more likely to be motivated to improve their lifestyle for disease prevention [24, 25]. It also suggests that middle-aged and older people in the UK are largely inclined to take actions to potentially reduce their dementia risk if external cues are provided, such as relevant information and preventive healthcare support.

There is a current dearth of research investigating the factors associated with individuals’ motivation to change lifestyle for dementia risk reduction, but more research has been done about the motivation to change lifestyle for the prevention of other conditions. Such studies have shown that motivation levels are highly associated with sense of responsibility, healthy lifestyle and adherence to treatment in individuals living with heart conditions and diabetes, for example [26–28]. Motivation levels thus appear to be associated with other factors that lead to better lifestyle choices and therefore have important implications to the prevention of multi-causal diseases, such as dementia.

Comparisons between motivation to change lifestyle in dementia and in other chronic diseases should be made with caution. The biopsychosocial impact of disease on the health of the individual will vary from person to person and overtime, impacting on motivation to change and lifestyle choices. Important differences might also exist between motivation to change behaviour for prevention of diseases vs. control of existing ones when individuals are currently living with the condition and its symptoms. Nevertheless, lifestyle-related risk factors for dementia are many (e.g. diabetes, obesity and high blood pressure) and dementia risk scores are based on shared attributable risk coefficients among these factors [1]. Considering that, future research exploring individuals’ motivation to change lifestyle for dementia risk reduction could draw upon the findings of studies involving other chronic conditions to further explore the dementia-related motivation to improve lifestyle. In addition, as the MOCHAD-10 scale measures general motivation to change lifestyle for dementia risk reduction (as opposed to specific risk factors), it may help inform multi-modal approaches to dementia risk reduction, particularly at primary health care services.

This study benefits from a large sample size, but the participants were likely to be individuals with an interest in dementia and how they may reduce their own risk of dementia in the future. Moreover, our sample differed from the characteristics of the average UK population in many aspects. For example, compared to the census of the Office for National Statistics (ONS) 2017/2018 [29], we had proportionally more people from England than from Wales and Northern Ireland (90.8% in our sample vs. 84.2% in the ONS data). We also had a higher proportion of women (72.9% in our sample vs. 50.7% in the ONS data), and more people aged 50–59 and 60–69 than 70+ (respectively: 40.5, 39.7, 19.8% in our study vs. 35.5, 31.21, 33.78% in the ONS data). Participants in this sample were also highly educated (*n* = 2297; 58.2%) which may have been due to the survey being shared across by the researchers’ university links, as well as other online platforms. This may have excluded those with lower health literacy, for example, who would potentially be less likely or less motivated to change their health behaviour. The use of both EFA and CFA is likely to have reduced such confounds, but future research should attempt to tap into participants often deemed difficult to reach in the research community.

The online nature of the study allowed for the collection of a large data set over a short period of time, but this method did not support the collection of data to assess convergent validity and divergent/ discriminant validity and test re-test reliability. Although such properties have been validated in the original Australian version of the scale [5], future work will test these aspects using the newly developed 10-item scale. Future studies using the MOCHAD-10 should also measure variance across gender and age. Furthermore, the way the online survey was set up meant there was no missing data as participants had to provide an answer to every item. This could mean we did not pick up on potential problems in the assessment of one or more items in terms of appropriateness or difficulty. Finally, as we only conducted quantitative psychometric validation of the scale, we did not tap into possible face validity issues in terms of the appropriateness or difficulty of items in the UK. It is hoped that the large sample sized mitigates some of these limitations, but future studies addressing such issue would be useful.

## Conclusions

This study investigated the factor structure and reliability scores of the first scale to measure attitudes and motivation to change lifestyle for dementia risk reduction for use in the UK. The newly validated version (MOCHAD-10) is a much shorter tool, but with a balanced number of items shared across the two retained factors. The overall moderate to high factor loadings and reliability scores demonstrate the robustness of the tool. The tool is suitable for use in clinical practice and research to measure motivation for lifestyle change to potentially reduce the risk of dementia and to implement relevant dementia risk reduction interventions.

## Abbreviations

CFA: Confirmatory Factor Analysis
CFI: Comparative Fit Index
EFA: Exploratory Factor Analysis
GFI: Goodness-of-Fit Index
KMO: Kaiser-Meyer-Olkin
MCLHB-DRR: Motivation to Change Lifestyle and Health Behaviours for Dementia Risk Reduction Scale
MOCHAD-10: Motivation to Change Behaviour for Dementia Risk Reduction Scale
ONS: Office for National Statistics
PAF: Principal Axis Factoring
RMR: Root Mean Square Residual
RMSEA: Root Mean Square Error of Approximation
